# Reduced glomerular size selectivity in late streptozotocin-induced diabetes in rats: application of a distributed two-pore model

**DOI:** 10.14814/phy2.12397

**Published:** 2015-05-25

**Authors:** Loay Lubbad, Carl M Öberg, Subramanian Dhanasekaran, Abderrahim Nemmar, Fayez Hammad, Javed Y Pathan, Bengt Rippe, Omran Bakoush

**Affiliations:** 1 Department of Surgery, College of Medicine & Health Sciences, United Arab Emirates UniversityAl Ain, United Arab Emirates; 2 Department of Nephrology, Clinical Sciences Lund, Lund UniversityLund, Sweden; 3 Department of Pharmacology and Therapeutics, College of Medicine & Health Sciences, United Arab Emirates UniversityAl Ain, United Arab Emirates; 4 Department of Physiology, College of Medicine & Health Sciences, United Arab Emirates UniversityAl Ain, United Arab Emirates; 5 Department of Internal Medicine, College of Medicine & Health Sciences, United Arab Emirates UniversityAl Ain, United Arab Emirates

**Keywords:** Capillary permeability, diabetic nephropathy, fractional clearance, glomerular filtration barrier, macromolecules, proteinuria, sieving coefficient

## Abstract

Microalbuminuria is an early manifestation of diabetic nephropathy. Potential contributors to this condition are reduced glomerular filtration barrier (GFB) size- and charge selectivity, and impaired tubular reabsorption of filtered proteins. However, it was recently reported that no significant alterations in charge selectivity of the GFB occur in early experimental diabetic nephropathy. We here aimed at investigating the functional changes in the GFB in long-term type-1 diabetes in rats, applying a novel distributed two-pore model. We examined glomerular permeability in 15 male Wistar rats with at least 3 months of streptozotocin (STZ)-induced diabetes (blood glucose ∼20 mmol/L) and in age-matched control rats. The changes in glomerular permeability were assessed by determining the glomerular sieving coefficients (*θ*) for FITC-Ficoll (molecular radius 20–90 Å) using size exclusion HPLC. The values of *θ* for FITC-Ficoll of radius >50 Å were significantly increased in STZ-diabetic rats compared to age-matched controls (*θ* for 50–69 Å = 0.001 vs. 0.0002, and *θ* for 70–90 Å = 0.0007 vs. 0.00006, *P* < 0.001), while *θ* for FITC-Ficoll <50 Å tended to be lower in diabetic rats than in controls (*θ* for 36–49 Å = 0.013 vs. 0.016, ns). According to the distributed two-pore model, there was primarily an increase in macromolecular transport through large pores in the glomerular filter of diabetic rats associated with a loss of small-pore area. Deterioration in the glomerular size selectivity due to an increase in the number and size-spread of large pores, with no changes in the permeability of the small-pore system, represent the major functional changes observed after 3 months of induced experimental diabetes.

## Introduction

Diabetic kidney disease (DKD) is a long-term complication of diabetes and is considered the worldwide leading cause of end-stage kidney disease and its related mortality (CDC 2014). Overt DKD is characterized by a progressive increase in urinary albumin excretion and a progressive decline in glomerular filtration rate (GFR). By contrast, early DKD is manifested by an increased GFR, and, as the disease progresses, development of microalbuminuria. Typically, the onset of microalbuminuria occurs before any decline in kidney function is observed.

The glomerular filtration barrier (GFB) comprises a series of layers, including the glomerular endothelium and the epithelial cells with their glycocalyx covers, and sandwiched in between, the glomerular basement membrane (GBM) (Haraldsson et al. 2008; Haraldsson and Nystrom 2012). Podocytes embrace the capillaries and stabilize the GFB as a whole. The sieving characteristics of the GFB are selective not only for molecular size but also for shape, deformability, and charge (Venturoli and Rippe 2005; Öberg and Rippe 2013, 2014). The changes in size selectivity that occur when the glomeruli are injured by disease are usually characterized by an increase in the number of low-selective pathways (large-pores) in the filtration barrier. Pathophysiological changes in the small-pore system are rarely seen and are usually accompanied by a marked increase in large-pore permeability (Öberg and Rippe 2014). The charge selectivity of the GFB is probably related to the presence of sulfated glycosaminoglycan (GAG) side chains of heparan sulfate (HS) proteoglycans in the anionic glycocalyx of podocytes and endothelial cells (van den Born et al. 2006; Öberg and Rippe 2013, 2014). Although the concept of charge selectivity of the GFB has been recently debated, a surface charge density of −5–20 mC/m^2^ is thought to be sufficient to prevent albuminuria (across high selectivity pathways) in normal conditions (Axelsson et al. 2011; Öberg and Rippe 2013).

Micropuncture studies have indicated that in normal conditions the albumin concentration in the glomerular filtrate is low (4–23 mg/L), consistent with a glomerular albumin sieving coefficient of 0.0001–0.0006 (Haraldsson et al. 2008; Haraldsson and Nystrom 2012). The pathophysiology underlying albuminuria in DKD has been extensively investigated by measuring the clearance of dextran macromolecules of graded size (30–60Å) (Lemley et al. 2000). However, because they are flexible, dextran molecules behave as random coils, facilitating their transglomerular permeability, which means that the glomerular sieving coefficient versus molecular radius could have been greatly overestimated (Michels et al. 1982).

Recent studies indicate that Ficoll macromolecules give more accurate estimates of protein glomerular sieving than dextran, and that a reduction in glomerular charge selectivity has been predicted to occur following acute renal ischemia–reperfusion injury and in the experimental diabetes model of nonobese diabetic (NOD) mice (Rippe et al. 2006; Barton 2008; De Carvalho et al. 2011; Yuen et al. 2012). However, studies from our group could not detect any significant alterations in GFB charge density after 3 and 9 weeks of STZ-induced diabetes in rats (Rippe et al. 2007). Data from Andersen et al. (2000), who studied glomerular Ficoll sieving in early human diabetic nephropathy also did not point to a major decline in the GFB charge selectivity, but demonstrated a decrease in size selectivity, that is, an increase in large-pore number in this disorder, although the sensitivity of their technique did not allow measurements of glomerular sieving coefficients to Ficoll molecules >60 Å in radius. A change in size selectivity in diabetic rats was also found in the pioneering studies of Remuzzi et al. (1993).To evaluate the functional alterations of the GFB in long-standing diabetes, in this study, we extended our previous investigations of experimental diabetes in rats from 2 to 3 months, again using a wide range of Ficoll macromolecular probes (Ficoll, molecular radii of 20–90 Å). In addition, a recently developed distributed two-pore model was used to analyze the sieving data.

## Material and Methods

Experiments were performed on 15 male Wistar rats with STZ-induced diabetes and 14 age-matched normal control rats. They were fed a standard rat chow and had free access to water. The experimental protocol was approved by the local animal research ethics committee (A19/11).

### Diabetic animals

The animals were made diabetic at the age of 8 weeks (weight 209–247 g) by intraperitoneal injection of streptozotocin (90 mg/kg; Biochemica, Sigma-Aldrich, Denmark). Rats with nonfasting plasma glucose concentrations >18 mmol/L were classified as diabetic and were given subcutaneous insulin injections daily (0.5 IU insulin; Act-rapid, 100 UI/mL; Novo Nordisk, Copenhagen, Denmark) to maintain blood glucose levels at 18–25 mmol/L. Rats with a plasma glucose concentration >17 mmol/L were given an additional 0.1 IU of insulin on the following day, whereas rats with a glucose concentration <13 mmol/L were given 0.1 IU less. After 3 months of diabetes, urine was collected and glomerular perm selectivity using FITC-Ficoll assessed.

### Creatinine clearance and urine protein concentration

Using metabolic cages, urine samples were collected over at least 12 h for measurement of creatinine, albumin, and gamma-glutamyl transpeptidase (GGT). The results of urinary albumin were expressed as grams of albumin per mole of creatinine in order to match the levels of albumin to the level of urine concentration. A blood sample was collected on the day of surgery for measurement of serum creatinine, urea, and albumin. All measurements were performed using a Cobas Integra 400 plus analyzer (Roche Diagnostics^®^, Basel, Switzerland). Creatinine clearance was calculated by the standard formula, in which the excretion of creatinine per minute [Urine creatinine × Vu (urine flow)] is normalized to plasma creatinine. Fasting blood glucose was measured in the morning on the day of surgery using an Accu-Check Performa glucometer (Roche Diagnostics-Diabetes^®^, Basel, Switzerland).

### Experimental protocol

The animals were anesthetized by an intraperitoneal injection of 60 mg/kg pentobarbital sodium and placed on a heated board to maintain body temperature at 36.5–37.5°C. The surgical preparation included tracheostomy (PE-240 tube), cannulation of the tail artery (PE-50 cannula) for blood sampling and monitoring mean arterial pressure with a strain gage pressure transducer and a digital display amplifier system, and cannulation of the femoral vein (PE-50) for infusions. The left kidney was exposed through a midline abdominal incision and its upper ureter was cannulated with a polyethylene tubing (PE-10) for collection of urine into preweighed microcapped tubes. Blood was withdrawn for glucose determination, and 20 min were allowed for the animal to stabilize and recover from the surgery before starting the experiment.

### Molecular probes and their measurement

Size selectivity of the GFB was assessed by using an infusion of 0.9% NaCl containing fluorescently labeled Ficoll (FITC-Ficoll) as neutral probes in nine male rats with 3 months of diabetes. These probes are not reabsorbed in the proximal tubules, but excreted in the final urine at a concentration which is equal to the concentration in Bowman's space multiplied by the urine-to-plasma inulin concentration ratio.

### Preparation of the Ficolls

FITC-Ficoll was given as a bolus of 12 *μ*g of FITC-Ficoll 70 combined with 288 *μ*g of FITC-Ficoll 400 and 100 *μ*g of FITC-labeled inulin to achieve a broad spectrum of molecular sizes. This was followed by a 20-min infusion (3 mL/h) containing FITC-Ficoll 70 (0.30 *μ*g/min), FITC-Ficoll 400 (7.2 *μ*g/min), and FITC-inulin (6.25 *μ*g/min). Urine was collected for 5 min, during which one midpoint (2.5 min) blood sample was taken for FITC-Ficoll and hematocrit determinations. Finally, the animals were euthanized with an overdose of potassium chloride *i.v*. under deep anesthesia, and the left kidney was weighed.

### High performance liquid chromatography (HPLC)

Fractionation of plasma and urine samples was performed using a size exclusion HPLC system (Waters) with an Ultrahydrogel 500 column, 7.8 × 300 mm (Waters) and a guard column, 6 × 40 mm (Rippe et al. 2007). Briefly, the mobile phase (phosphate buffer with 0.15 mol/L NaCl, pH 7.4) was driven by a pump (Waters 616), and fluorescence was detected with a fluorescence detector with the excitation wavelength set at 492 nm and the emission wavelength at 518 nm (Waters 2475). Test samples were delivered to the system with an autoinjector (Waters 717plus). The system was controlled using Empower Pro software (Waters). The column was calibrated with five narrow Ficoll fractions (73, 59, 46, 38, 30 Å, respectively) and three dextran standards (80, 410, 670 Å, respectively) labeled with FITC. Albumin, alcohol dehydrogenase, IgM, and apoferritin were also used to calibrate the column and were detected with an UV absorbance detector (Waters 486). The void volume (*V*_0_ = 6.31 mL) and total volume (*V*_T_ = 11.16 mL) of the column were determined with blue dextran (2 × 10^6^ Da) and glycine (75 Da), respectively. The distribution coefficient (*K*_AV_) was then calculated using the formula *K*_AV_ = (*V*_e_ − *V*_0_)/(*V*_T_ − *V*_0_), where *V*_e_ is the elution volume of the solute and *V*_T_ is the total bead volume of the gel column measured by glycine.

### Calculations

The glomerular sieving coefficients (*θ*) for Ficoll were obtained by dividing the concentrations of Ficoll in the urine by their average plasma concentrations and the inulin urine to plasma concentration ratio.

### Pore theory mathematical model

The two-pore theory of neutral macromolecular transport across microvascular walls has been successfully used to describe the sieving of plasma solutes through systemic and glomerular capillary walls (Rippe and Haraldsson 1994; Bakoush et al. 2004; Rippe et al. 2006; Öberg and Rippe 2014). Here we used a distributed two-pore model (Öberg and Rippe 2014) for the theoretical analysis of the data. In this model, the size selective structures of the GFB are assumed to be log-normally distributed small-pore and large-pore populations. The distributed two-pore model is distinct from the log-normal (single) pore model (Remuzzi et al. 1993), or particularly, the log-normal + shunt distributed model (Remuzzi et al. 1993; Andersen et al. 2000), in that the “shunt” concept (pores of infinite radius) is here abandoned. Compared to the classic two-pore model (having fixed pore sizes), the distributed two-pore model provides a better fit of Ficoll sieving data, especially in the region between the small-pore and the large-pore sieving curves, that is, at molecular radii between of 45–65 Å (Öberg and Rippe 2014).

The major parameters of the two-pore model are as follows: the mean small-pore radius (*r*_s_) and standard deviation (SD) (*s*_s_), the mean large-pore radius (*r*_L_) and SD (*s*_L_), the unrestricted pore area over unit diffusion path length (*A*_0_/Δ*x*), and the fraction of the glomerular ultrafiltration coefficient accounted for by the large pores (*α*_L_). Ideally, these parameters describe the membrane properties without being influenced by hemodynamic factors. *A*_0_/Δ*x* is a “diffusive parameter” that reflects the unrestricted surface area (*A*_0_) and the “effective” thickness (Δ*x*) of the GFB. The parameter *α*_L_ reflects the abundance of large pores in the glomerular filter and is calculated from the fraction of GFR that is diverted through the large pores (*J*v_L_/GFR). The nonequality between *f*_L_ (JvL/GFR) and *α*_L_ is due to the plasma oncotic pressure in the glomerular capillary plasma, which is about 28 mm Hg during normal conditions (Öberg and Rippe 2014).

### Statistical analysis

The data are expressed as mean ± SEM or as median and interquartile range. Data from control and experimental groups were compared using Student's *t*-test, and Mann–Whitney nonparametric test where appropriate. A *P*-value <0.05 was considered significant.

## Results

The baseline characteristics of the rats are listed in Table1. There was no statistical difference in serum creatinine levels between diabetic and age-matched control rats. Rats that had been diabetic for three months had lower body weights (*P* = 0.02), larger kidneys (*P* = 0.001), lower serum albumin (*P* = 0.003), twofold higher albumin excretion (*P* = 0.02) and 50 times higher GGT excretion (*P* < 0.001) in urine.

**Table 1:** Baseline characteristics of control rats and diabetic rats given as mean ± SE or median (interquartile range).

Variable	Control	Diabetic	*P*-value
Number	14	15	
Body weight (g)	316 ± 8.3	286 ± 9.4	0.02
Kidney weight (g)	0.91 ± 0.02	1.02 ± 0.01	0.001
Blood glucose (mmol)	5.5 ± 0.5	19.9 ± 1.2	<0.001
MAP (mmHg)	109 ± 3.5	108 ± 4.4	0.44, ns
Serum creatinine (*μ*mol/L)	18.3 ± 0.6	19.3 ± 0.8	0.54, ns
LDH (U/L)	97 (119)	80 (59)	0.13, ns
Serum albumin (g/L)	37.4 ± 0.62	34.3 ± 0.63	0.003
Albuminuria (mg 24 per hour)	60.8 (76)	115 (41)	0.02
U-GGT U/L	0.9 (0.8)	48 (140)	<0.001

MAP, mean arterial blood pressure; LDH, lactate dehydrogenase; U-GGT, urine gamma-glutamyl transpeptidase.

**Figure 1:**
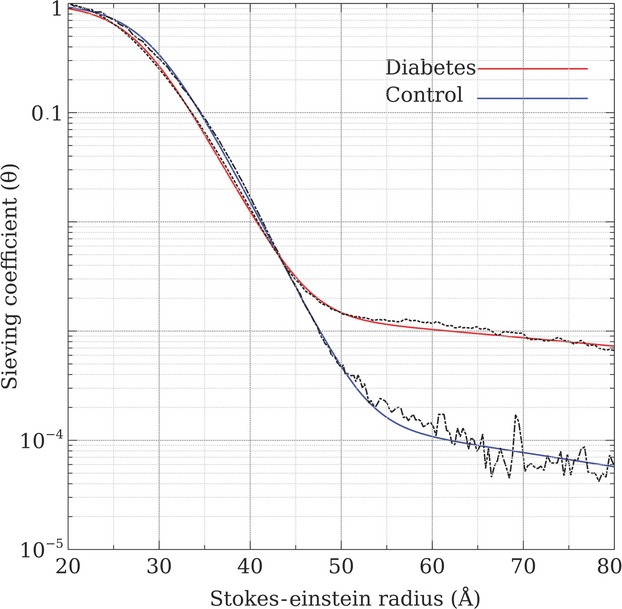
The increased permeability sieving coefficients of FITC-Ficoll in rats with 3 months of STZ-induced diabetes compared to age-matched control rats.

Table2 and Figure1 show the values of theta (*θ*) for FITC-Ficoll molecules in STZ-diabetic rats compared to age-matched controls. The sieving coefficients for Ficoll macromolecules in the molecular range of >50 Å were significantly increased in diabetic rats compared to age-matched control rats (*θ* for Å 50–69 = 0.001 vs. 0.0002, and *θ* for Å 70–90 = 0.0007 vs. 0.00006, *P* < 0.001). The sieving coefficients for FITC-Ficoll in the molecular range of <50 Å tended to be lower in diabetic rats (*θ* for Å 36–49 = 0.013 vs. 0.016, ns).

**Table 2:** Sieving coefficients for FITC-Ficoll (20–90 Å) in diabetic and age-matched control rats

Size (Å)	<20	(20–35)	(36–49)	(50–69)	(70–90)
Control	1.00	50.04 × 10^−2^	16.36 × 10^−3^	2.24 × 10^−4^	5.87 × 10^−5^
Diabetes	1.00	46.04 × 10^−2^	12.74 × 10^−3^	11.93 × 10^−4^	71.83 × 10^−5^

### Two-pore modeling

**Figure 2:**
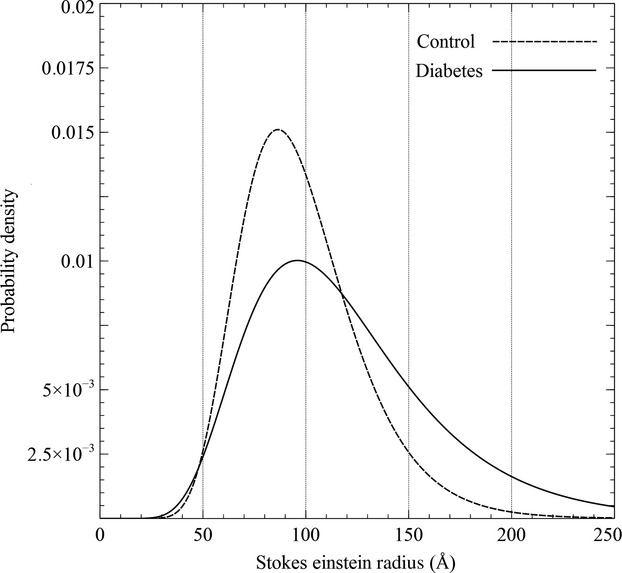
The change in the distribution of the large-pore radius in the GFB occurring in rats with 3 months of STZ-induced diabetes compared to age-matched control rats.

The best-curve fits of *θ* values versus Stokes-Einstein molecular radii (*a*_e_) for Ficoll according to the two-pore model were obtained using the parameters for nine diabetic animals and six healthy controls (Table3). The fractional large-pore hydraulic conductance (*α*_L_) increased ninefold in diabetic animals relative to controls (*P* < 0.05). Similarly, the fractional large-pore volume flux (*J*_vL_/GFR) was sixfold higher in diabetic animals (*P* < 0.01). Furthermore, the large-pore distribution was wider in diabetic animals (Fig.2). As expected, the small-pore radius (*r*_S_) did not change, but the effective pore area over unit diffusion path length (*A*_0_/Δ*x*) was lower in diabetic animals. Thus, the increase in the *θ* of Ficoll of radius >50 Å was primarily due to an increase in macromolecular transport through large pores in the glomerular filter. The reduced *θ* for small Ficoll macromolecules in diabetic rats was primarily due to a loss of small-pore surface area.

**Table 3:** Distributed two-pore parameters for FITC-Ficoll

Model parameter	Control (*N* = 6)	Diabetic (*N* = 9)
Small-pore radius (*r*_S_), Å	37.1 ± 1.1	37.5 ± 0.5
Small-pore spread (*s*_S_)	1.15 ± 0.01	1.14 ± 0.00
Large-pore radius (*r*_L_), Å	94 ± 8	111 ± 9
Large-pore spread (*s*_L_), Å	1.34 ± 0.01	1.47 ± 0.02*
*A*_0_/Δ*x* × 10^5^, cm†	17 ± 5	8 ± 2
L_p_S, mL/min/mmHg†	0.38 ± 0.09	0.19 ± 0.05
*α*_L_ × 10^5^	5 ± 2	44 ± 13*
*J*_vL_/GFR × 10^5^	32 ± 8	199 ± 45**
Volume recirculation (*J*_vL,iso_), *μ*L/min†	0.3 ± 0.1	1.5 ± 0.7
*A*_0,L_/A_0_ × 10^6^	8 ± 3	44 ± 21
GFR, mL/min†	1.20 ± 0.2	0.75 ± 0.2
Goodness of fit, *χ*^2^	0.25 ± 0.06	0.18 ± 0.02

*A*_0_/Δ*x* effective pore area over unit diffusion path length in centimeters; *A*_0,L_/*A*_0_ fractional large-pore area; L_p_S hydraulic conductance (calculated from *A*_0_/Δ*x*); *α*_L_ fractional large-pore hydraulic conductance; *J*_vL_/GFR fractional large-pore volume flux; *χ*^2^ = “Goodness of fit”.

* *P* < 0.05

** *P* < 0.01.

† Refers to both kidneys.

## Discussion

We investigated the functional changes in the GFB after 12 weeks of diabetes duration in an STZ-diabetic animal model. Our results show that in advanced DKD, size selectivity of the GFB declines. Advanced diabetes was associated with a substantial rise in the clearance of Ficoll macromolecules of molecular radius >50 Å. According to the distributed two-pore model of macromolecular transport, this rise reflects a predominant increase in the number and/ or size of large pores in the GFB, with no or little effect on the small-pore pathway.

In STZ-diabetic rats, disruption to GFB integrity is expected to start within 2 months of hyperglycemia due to high glucose as such (*via* e.g., advanced glycation end products), inflammatory cytokines, reactive oxygen species, activation of the renin–angiotensin system, systemic endothelial dysfunction, and loss of podocytes and their function (Barton 2008; Yuen et al. 2012). Advanced DKD is also associated with development of tubulo-interstitial fibrosis and tubular dysfunction, as indicated by urinary tubular markers such as increased urinary excretion of GGT (Rippe et al. 2007; De Carvalho et al. 2011).

The GFB separates molecular species in the circulation depending on their size, charge and conformation (Venturoli and Rippe 2005). In our study, the small-pore system was apparently unaffected in late STZ diabetes, except for a smaller surface area in the GFB, which could be partly explained by the lower GFR in the diabetic animals (0.75 mL/min) compared to controls (1.20 mL/min). Thus, in contrast to the large-pore system, size selectivity of the small-pore system did not seem to change. However, an initial decrease in the size selectivity of the small-pore system might be reversible, and if such a change had occurred, it would have gone unnoticed in our setup. Indeed, if such a decrease in selectivity had occurred, it would help explain the isolated albuminuria commonly observed in the clinic in early DKD. Another possibility, frequently suggested in the literature, is that the selective albuminuria commonly observed in early DKD be due to a selective decrease in the proximal tubular reabsorption of anionic macromolecules (cf. albumin), or to a decreased charge selectivity of the GFB, which would, if affecting a highly size-selective barrier, lead to isolated albuminuria (see below). However, in a previous rat experiment from this group, there were no signs of any alterations in charge selectivity of the GFB after three and 8 weeks of diabetes duration (Rippe et al. 2007).

It has been hypothesized that the effective radius of the size selective elements in the GFB is about 37 Å, which is very close to the effective radius of albumin (~35.5 Å) (Öberg and Rippe 2014). Thus, even a small negative charge of 5–20 mC/m^2^ (Öberg and Rippe 2013) will completely block albumin from entering the small-pore pathway. This relatively small charge has been estimated to add about ~1.5 Å to the radius of albumin (36 Å) and to subtract about ~1.5 Å from the small-pore radius, effectively blocking albumin from entering the small-pore pathway. Consequently, if the charge barrier is damaged, a negatively charged molecule the size of albumin can enter the small-pore pathway, leading to a substantial increase in its fractional clearance compared to the increase for the neutral molecule of the same size. On the other hand, an increase in the number or size/spread of the large pores will always allow the transport of molecules much larger than albumin irrespective of charge selectivity. Thus, our results support the notion that a dysfunction primarily of the size-restrictive properties of GFB plays a role in advanced STZ diabetes. This is consistent with the increase in urinary excretion of large proteins such as IgM (molecular radius 120 Å) in patients with DKD, which could only be attributed to deterioration of the size selectivity of the GFB and the development of “shunt” pathways (Bakoush et al. 2002).

Our data are consistent with the glomerular Ficoll sieving data of Remuzzi et al. (1993) in rats and those of Andersen et al. (2000), measured in early stages of human diabetic nephropathy. In the latter study DKD was associated with a tenfold increase in the number of large glomerular pores, while the small-pore system was largely unchanged. Treatment with Losartan, an angiotensin II receptor blocker, partly reduced this alteration in size selectivity. The present study not only conforms to the study of Andersen et al., but also extends it by investigating glomerular Ficoll sieving coefficients for molecular radii exceeding 60 Å. Furthermore, in light of the documented “hyperpermeability” of Ficoll versus proteins in the molecular size (radius) interval of 20–45 Å (Asgeirsson et al. 2007), the discrepancy in *θ* for Ficoll_36Å_ (*θ* ~0.1) and that for native albumin (*θ* ~0.0001), in the study of Andersen et al., can now be explained without invoking charge-dependent restriction of albumin (Venturoli and Rippe 2005; Öberg and Rippe 2014).

The present study also explains why older dextran-based studies did not reveal any changes in size selectivity in diabetic animals. Michels et al. (1982) studied the changes in the glomerular permeability occurring in late diabetes using neutral dextran. The study, however, failed to detect any alterations in the size selectivity of the diabetic GFB. Studies by us and others have demonstrated that dextran molecules show an increased molecular “extension” (larger SE-radius for any given MW) and thereby, conceivably, an increased “flexibility” (Venturoli and Rippe 2005) than, for example, proteins of equal MW. This increased flexibility of dextran molecules seems to make dextran hyperpermeable across the GFB. Hence, dextran molecules may assume radii which sometimes are essentially smaller than corresponding to their measured SE-radius. Thus, dextran may actually pass through pores which are smaller than their chromatographic SE-radius, that is, through pores which would be inaccessible by hard spheres of equal molecular radius. Ficoll molecules, in this sense, deviate much less from the behavior of hard spheres (or proteins), and has by our group been shown to function as rather exact surrogate markers for protein permeability in the size range of 50–80 Å. For that reason, large Ficoll molecules are more suitable as probes for assessing size selectivity caused by alterations in the number (or size) of large pores in the GFB than dextran.

## Conclusion

In experimental diabetes, the glomerular clearance of large macromolecules is increased via an increased number of large pores and an increase in the spread of large-pore radius in the GFB, while the contributions of changes in the small-pore system or reductions in charge selectivity appear negligible.
